# Estimating the productivity cost of administrative duties and work-related engagements from submission trends during COVID-19 pandemic lockdown

**DOI:** 10.1128/mbio.01467-24

**Published:** 2024-06-18

**Authors:** Arturo Casadevall, Lorraine F. Clark

**Affiliations:** 1 Johns Hopkins Bloomberg School of Public Health, Baltimore, Maryland, USA; 2 American Society for Microbiology, Washington, DC, USA

**Keywords:** COVID-19, lockdown, publishing

## Abstract

During the initial months of the coronavirus disease 2019 pandemic, *mBio* experienced a large increase in the number of submissions, a phenomenon that was also observed for journals of different fields. Since most research laboratories were closed, this increase cannot reflect increased research activity. In this editorial, we propose that the increase in submissions reflected the release of a backlog of unpublished work following a reduction in work-related engagements including scientific travel, which in turn provides an estimate of the productivity costs of such activities on research output.

## EDITORIAL

As the worst days of the coronavirus disease 2019 (COVID-19) pandemic recede into the rear-view mirror, we can reflect on some of the experiences of that terrible time and how they affected science and scientific publishing. The COVID-19 pandemic was a tremendously disruptive event for humanity, and science was no exception.

An interesting phenomenon was a sharp increase in the number of articles submitted to *mBio* in the weeks following the mid-March 2020 lockdown when much of the world shut down non-essential operations to slow the spread of the pandemic. Submissions for the first and last 2 weeks of March 2020 were not very different, being 134 and 141 papers, respectively. However, when one considers the half year before and after March 2020 a different picture emerges. In the 6 months preceding March 2020, 1,440 papers were submitted to *mBio*, while in the succeeding 6 months, this number rose to 1,777, representing a 23.4% increase. Furthermore, in the months of April, June, and July of 2020, *mBio* received over 300 submissions each, representing the months with the highest submissions until that time. At the journal, the increase in submissions coupled with the disruptions following the transition to working remotely and the pandemic conditions required pivoting operations to maintain its publication tempo. The experience of *mBio* with increased submissions after COVID-19 lockdowns was mirrored by that of other journals in other fields including radiology (up 41.8%) (1), ecology (up 6.3%–27%) (2), food and agriculture (up 29%) (3), neurosurgery (up 55%–79%), geophysics (up 7%) (4), and pediatrics (up 61.6%). The fact that this phenomenon was observed across such diverse scientific fields implies a general effect on scientific productivity.

What could have accounted for the rise of submissions associated with the pandemic lockdown? Since most laboratories closed and *mBio* publishes predominantly experimental studies, the increase in submissions cannot be the result of additional research activities. Many scientists transitioned to working remotely from home, and in the absence of new data emanating from their laboratories, we surmise that the increase in submissions reflected getting papers written and submitted based on work that had already been done. Hence, another outcome of the pandemic is an estimate of the body of unpublished data that needs to be written into papers and submitted for publication. The 23.4% increase in submissions following pandemic lockdowns may suggest that this backlog is as large as 20%–25% of the yearly scientific output of a typical microbiology laboratory, and the larger increases in other journals suggest that this backlog may be even greater in other fields. As students and postdoctoral fellows found themselves unable to go to the laboratory and generate new data, we suspect some of the increase in submissions could reflect that many refocused their energies on getting unpublished data prepared for publication.

While the rise in submissions reflected increased scientific productivity in the sense that publications reflect the reporting of research, it is noteworthy that lockdowns and the transition to remote work were often associated with much more difficult working conditions (5). Several studies have documented how the COVID-19 pandemic had detrimental effects on science and women scientists (6), particularly among those who had to care for young children at home (7). Consistent with this, the increased submissions during the initial stages of the pandemic were associated with fewer female authors perhaps reflecting disproportionate hardships for female scientists (7–9). Since teaching at many institutions transitioned from in-person classroom instruction to videoconferencing, one cannot attribute the increase in productivity to reduced teaching loads for academics. Pivoting from in-class to remote teaching required a considerable amount of work and adjustment for both teachers and students. For scientists involved in editorial activities, work simply moved remotely, as exemplified by the transition of the *Journal of Clinical Investigation* weekly editorial meetings from in-person to videoconference (10). Hence, it is difficult to attribute this increase in scientific productivity to the shift from in-person to remote work or better working conditions.

One activity that abruptly ended during the pandemic was travel related to scientific activities as all in-person conferences and seminars were canceled and most moved to a remote format. For example, ASM canceled the in-person Microbe meeting in both 2020 and 2021 and transitioned these to fully online meetings before Microbe returned to an in-person format in 2022. Commuting to work ceased, and administrative work, departmental meetings, and seminars were reduced. Could it be that when scientists had to forgo administrative duties and work-related engagements, such as traveling to conferences and seminars, they found the time to write more papers by remaining at home, even though pandemic conditions brought their own new sets of difficulties?

For many scientists, the ability to travel to conferences and meet with colleagues is a desirable and essential aspect of scientific life. Scientific travel is justified based on the need to present one’s work and learn new scientific findings, meet and establish social connections with other scientists, and facilitate collaborations. Many scientists consider scientific travel essential for meeting colleagues and scientific advancement (11). Travel is particularly important for younger scientists to create their networks, meet established scientists in their field, and become known to colleagues. However, there is seldom discussion of the costs, if any, of scientific travel to the enterprise of science, but this may be changing. With rapid global warming, some scientists are increasingly concerned about the carbon footprint of scientific travel, which can range from 1 to 3 tons of carbon per meeting attendee (12, 13). Furthermore, virtual conferences or meetings that offer both in-person and online components can provide an opportunity for expanded and diversified attendance, for example, travel can be challenging for those with disabilities and international scientists for whom overseas travel is not always financially viable (14).

A certain amount of travel to meet colleagues, secure collaborations, and attend conferences is almost certainly essential for most scientists. However, like most things in life, there is probably a point after which smaller benefits are derived from more frequent travel, and excessive travel can be counterproductive since prolonged absences from laboratories can undermine the supervision of trainees and laboratory work. Travel takes time, and the time required to prepare for a conference (obtaining visas, booking travel, creating slide decks, arranging meetings with collaborators), board planes, clear customs, and adapt to new settings does not usually translate into scientific productivity. When and where scientific travel transitions from benefit to debit are likely to differ for individual scientists depending on where they are in their careers, and we have no advice on that regard. When scientists make their individual travel cost-benefit calculations, they may consider productivity effects in addition to the carbon footprint of the journey.

In addition to travel, scientists also spend much of their work time on administrative and departmental duties, including attending meetings and seminars, taking guest speakers out for meals, chatting with colleagues in their offices, over coffee, and in the hallways, and commuting to and from work. While many of these activities are pleasant social aspects of scientific life, these commitments either ceased entirely or were drastically reduced after March 2020 until workplaces gradually reopened. This likely also contributed to the increase in manuscript submissions as researchers were largely freed from various tasks that are usually part of their workday.

One might argue that the increase in submissions to *mBio* could be due to the volume of manuscripts the journal received related to severe acute respiratory syndrome coronavirus 2 (SARS-CoV-2)/COVID-19 during and after March 2020, considering research activities in this area ramped up dramatically after the discovery of the virus. Therefore, we looked more closely at submissions to the journal from 1 March 2020 through 31 August 2020. As stated earlier, the journal received 1,777 submissions during this time, and we found 152 of these focused on SARS-CoV-2/COVID-19. Subtracting this from the total number of manuscripts, the journal received in those 6 months yields 1,625 submissions, which still represents a 12.8% increase compared to the 6 months leading up to March 2020. Therefore, our hypothesis that the halting of conference travel and reduced administrative burdens may have provided scientists with the time to catch up on writing and completing manuscript drafts is still supported. Furthermore, the additional 152 manuscripts *mBio* received on SARS-CoV-2/COVID-19 after 1 March 2020 may have been enabled by the scientists’ time getting shifted from work-related travel and administrative duties to focusing on this novel virus. Thus, the shift in how researchers spent their work-related time after the 2020 lockdown may have not only supported the increase in submissions to *mBio* in general but also the growth in submissions on SARS-CoV-2/COVID-19. Once universities welcomed students back in the fall of 2020 (15) and travel restrictions were lifted by the United States in November 2021 (16), submissions to the journal decreased to 1,621 between September 2020 and February 2021 and to 1,605 between November 2021 and May 2022, potentially suggesting that the re-uptake of administrative tasks and travel reduced the time researchers had to focus on writing manuscripts.

Another point to consider is whether the increase in submissions to *mBio* was due to more submissions of reviews and perspectives. Indeed, the journal observed a significant increase in those article types. In the 6 months leading up to March 2020, 23 minireviews and 2 perspectives were submitted. This drastically changed upon lockdown as the numbers rose to 72 minireviews and 24 perspectives between March and August of 2020, representing a change of 213% for minireviews and 1,100% for perspectives. Of the combined 96 minireviews and perspectives submitted between March and September of 2020, 37 dealt with the subject of COVID-19/SARS-CoV-2, indicating researchers’ focus shifting to the novel virus enabled by the suspension from travel, commuting, and administrative tasks as was the overall rise in minireviews and perspectives.

The COVID-19 pandemic undoubtedly changed our world profoundly and challenged various ways in which we traditionally thought and operated. One aspect in this regard is the amount of time scientists spent traveling to conferences and carrying out administrative duties prior to 2020 and how this may have affected their publication activity. The shift in how researchers worked after March 2020 most certainly enabled scientists to focus on their manuscript backlog, write reviews and perspectives, and/or shift their research to the novel virus. Restrictions introduced during 2020 largely eased toward the end of 2021, allowing researchers to return to their laboratories, which means a return to commuting and conference travel. However, researchers may want to consider whether there is a sweet spot in terms of the number of administrative engagements and conferences one should take on while remaining productive in their research in a way that avoids buildup of unpublished results. While it is essential that researchers support their institutions through their involvement in administrative tasks and present cutting-edge research results at meetings, it is also crucial that scientific results are published in a timely manner to establish precedence and communicate findings to the broader community.
